# Filamin A Is Required for NK Cell Cytotoxicity at the Expense of Cytokine Production *via* Synaptic Filamentous Actin Modulation

**DOI:** 10.3389/fimmu.2021.792334

**Published:** 2022-01-04

**Authors:** Nayoung Kim, Eunbi Yi, Soon Jae Kwon, Hyo Jin Park, Hyung-Joon Kwon, Hun Sik Kim

**Affiliations:** ^1^ Department of Convergence Medicine, Asan Institute for Life Sciences, Asan Medical Center, University of Ulsan College of Medicine, Seoul, South Korea; ^2^ Department of Biomedical Sciences, Asan Medical Center, University of Ulsan College of Medicine, Seoul, South Korea; ^3^ Stem Cell Immunomodulation Research Center (SCIRC), University of Ulsan College of Medicine, Seoul, South Korea

**Keywords:** NK cells, filamin A, filamentous actin, cytotoxicity, cytokine, immune synapse

## Abstract

Natural killer (NK) cells are innate cytotoxic lymphocytes that efficiently eliminate malignant and virus-infected cells without prior activation *via* the directed and focused release of lytic granule contents for target cell lysis. This cytolytic process is tightly regulated at discrete checkpoint stages to ensure the selective killing of diseased target cells and is highly dependent on the coordinated regulation of cytoskeletal components. The actin-binding protein filamin crosslinks cortical actin filaments into orthogonal networks and links actin filament webs to cellular membranes to modulate cell migration, adhesion, and signaling. However, its role in the regulation of NK cell functions remains poorly understood. Here, we show that filamin A (FLNa), a filamin isoform with preferential expression in leukocytes, is recruited to the NK cell lytic synapse and is required for NK cell cytotoxicity through the modulation of conjugate formation with target cells, synaptic filamentous actin (F-actin) accumulation, and cytotoxic degranulation, but not granule polarization. Interestingly, we also find that the loss of FLNa augments the target cell-induced expression of IFN-γ and TNF-α by NK cells, correlating with enhanced activation signals such as Ca^2+^ mobilization, ERK, and NF-κB, and a delayed down-modulation of the NKG2D receptor. Thus, our results identify FLNa as a new regulator of NK cell effector functions during their decision to kill target cells through a balanced regulation of NK cell cytotoxicity vs cytokine production. Moreover, this study implicates the cross-linking/bundling of F-actin mediated by FLNa as a necessary process coordinating optimal NK effector functions.

## Introduction

Natural killer (NK) cells are a unique subset of effector lymphocytes that provide innate surveillance against tumors and virus infections (1–4). The effector functions of NK cells are rapidly mediated by direct cytolysis of target cells *via* the polarized secretion of preformed cytotoxic granules in addition to cytokine production such as IFN-γ. The NK cell decision to kill target cells is highly regulated and is dependent on integrated signals from a multitude of activating and inhibitory receptors that enable the selective killing of diseased cells over normal healthy cells (5, 6). Central to this process is the formation of a lytic immunological synapse (IS) between NK cells and target cells, which secures specific targeting of cytolytic process to a bound target cell. The creation of mature lytic IS requires the coordinated regulation of cytoskeletal components, signaling molecules, and cellular organelles (7, 8) and progresses *via* discrete regulated steps that function as stepwise checkpoints in accessing NK cell-mediated cytolysis (9).

The accumulation of filamentous actin (F-actin) at the lytic IS is the major cytoskeletal reorganization event and is critical to integrin-mediated adhesion and cytotoxicity (10, 11). F-actin polymerization in NK cells is also dynamically regulated downstream of the activating and inhibitory receptors (12–14). For example, the engagement of the activating receptor NKG2D promotes the assembly of NKG2D microclusters into a ring-shaped structure at the center of the lytic IS, F-actin polymerization, NK cell adhesion to target cells, and cytotoxicity (13, 15, 16). F-actin networks also need to be disassembled to form a conduit, and lytic granules have access to the plasma membrane through this conduit for the release of their contents toward the bound target cell (8, 17, 18). Prior to this process, lytic granules are required to be converged around the microtubule organizing center (MTOC) by a microtubule minus-end-directed motor complex and then be delivered to the NK-target cell interface along with the MTOC (8, 19). This coordination between the actin and tubulin cytoskeletons leads to the directed secretion of lytic granule contents and stimulatory cytokines at the lytic IS to promote target cell death.

Actin cytoskeleton remodeling is mediated by a large number of actin-binding proteins, principally the actin-related protein 2 and 3 (Arp2/3) complex and the formins, which are the central nucleating factors (NFs) for this reorganization. Arp2/3 and the formin hDia1 have been shown to play differential roles in NK cytotoxicity; Arp2/3 depletion impairs cell adhesion and actin assembly at the lytic IS, whereas hDia depletion leads to defects in lytic granule polarization and secretion (20). There are two major nucleation promoting factors (NPFs) for Arp2/3, Wiskott-Aldrich syndrome protein (WASp) and WAVE2 (21). F-actin reorganization at NK IS occurs downstream of activating receptor-mediated Vav1 activity and is dependent on WASp which promotes F-actin branching (8). WAVE2 induces NK cell function in IL-2-stimulated human NK cells in a WASp-independent manner, suggesting that WASp and WAVE2 activities define parallel pathways of F-actin reorganization and function (22). Moreover, the function of Arp2/3 in actin branching is antagonized by Coronin 1A which promotes localized F-actin disassembly to permit lytic granule release at the lytic NK IS (23). Accordingly, Coronin 1A deficiency impairs the delivery of lytic granules to the IS and thus suppresses NK cell cytotoxicity (23), suggesting that a dynamically regulated actin cytoskeleton is required to ensure selective cytolytic activity toward target cells.

In addition to actin polymerization, de-polymerization, and branching, bundling of polymerizing actin filaments is required for the formation of higher-order actin network, which has been implicated in cell migration and cell-cell interaction (24–26). F-actin filaments are collected into fiber bundles and cross-linked into a fiber meshwork through the binding of filamins (27). Filamin A (FLNa) is one of the isoforms that crosslinks F-actin to stabilize the actin filament networks and links them to cellular membranes (24, 28). Arp2/3 complex and FLNa localize to the junctions between orthogonally intersecting actin filaments. The actin network is organized by the Arp2/3 complex and maintained by FLNa (25). In T cells, FLNa is necessary for optimal integrin-induced force transmission, flow adhesion, and the trafficking of T cells (29). FLNa phosphorylation also controls TCR-induced LFA-1 activation (30). Hence, FLNa contributes to the proper functioning of T cells as a link between the integrins and the actin cytoskeleton (29). However, much less is known about the role of FLNa in the regulation of NK cell effector functions despite its important role in the reorganization of the actin cytoskeleton. In our current study, we sought to assess the contribution of FLNa to the effector functions of human NK cells with a focus on NK cell cytotoxicity.

## Materials and Methods

### Cell Culture

NKL cells, a human NK cell line (gift of M. Robertson, Indiana University), were cultured in RPMI1640 (Gibco) containing FBS (10%), sodium pyruvate (1 mM), and rIL-2 (200 U/mL). NK92 cells (gift of K. S. Campbell, Fox Chase Cancer Center) were cultured in α-MEM (Gibco) containing FBS (20%), MEM vitamin solution (1%; Gibco), 2-mercaptoethanol (0.1 mM; Gibco), and rIL-2 (200 U/mL). Myelogenous leukemia K562 (American Type Culture Collection), B-lymphoblastoid 721.221 (a gift of J. Gumperz and P. Parham, Stanford University, Stanford, CA), mouse mastocytoma P815 (American Type Culture Collection), and P815-ULBP1 (P815 with stable surface expression of ULBP1) cells (31) were cultured in IMDM medium (Hyclone) containing FBS (10%). K562 cells are known to express ligands for NKG2D, DNAM-1, and NKp30 receptors (32, 33), whereas the lysis of 721.221 cells are associated with 2B4, NKp44, and NKp46 receptors (34, 35). NK92 cells were transduced with a vector expressing the 176V variant (high affinity) of CD16, to generate CD16.NK92 cells. The chronic myeloid leukemia (CML)-blast crisis (BC) cell line KCL22M harboring tyrosine kinase inhibitor-resistant BCR-ABL1 T315I mutation (a gift of W. Y. Chen, Beckman Research Institute, City of Hope) were cultured in RPMI1640 (Gibco) medium containing FBS (10%) and Imatinib (2 μM; Santa Cruz). NKG2D, DNAM-1, and, to a lesser extent, NKp30 contribute to the lysis of KCL22M cells by NK cells (36). The cells used were confirmed to be mycoplasma-free.

Expansion of primary human NK cells were performed according to previous procedure (37), with minor modifications. Briefly, peripheral blood mononuclear cells (PBMCs) were isolated using lymphocyte separation medium (MP Biomedicals). PBMCs were co-incubated at 1.5 × 10^6^ cells/well in a culture plate (24-well) with irradiated K562-mb15-41BBL feeder cells (gift of D. Campana, National University of Singapore) at 1 × 10^6^ cells/well in Stem Cell Growth Medium (SCGM; CellGenix) containing FBS (10%) and rIL-2 (10 U/mL). The culture medium was changed every 2-3 days using fresh medium containing rIL-2. After 1 week, CD3-positive selection kit (Stem Cell Technologies) was used to deplete residual T cells. Thereafter, remaining cells were incubated in SCGM containing FBS (10%), rIL-2 (100 U/mL), and rIL-15 (5 ng/mL) for two extra weeks with a medium change every 2-3 days. The cells after expansion were assessed by flow cytometry as being 96-99% CD3-CD56+.

### Reagents

The following antibodies (Ab) were used to detect the indicated proteins: Isotype control mouse IgG1 (MOPC-21; Sigma), NKG2D (149810; R&D Systems), CD244/2B4 (C1.7; Beckman Coulter), actin (C4; BD Biosciences), and p-ERK1/2 (9101), ERK1/2 (9102), pS473-Akt (9271), and FLNa (4762) (all from Cell Signaling). The Abs used for flow cytometry included anti-CD107a-FITC (H4A3), anti-CD56-PE (NCAM16.2), and anti-CD3-PerCP (SK7) (all from BD Biosciences).The fluorochrome-conjugated Ab and reagents used for confocal microscopy were the Vybrant CFDA (CFSE) SE cell tracer kit (V12883), Cell tracker Orange CMTMR (C2927), Alexa Fluor 488 phalloidin (A12379) (all from Invitrogen), Alexa Fluor 647 anti-human perforin (308109; Biolegend), and anti-human FLNa (MAB1678; Chemicon). Secondary anti-mouse and anti-rabbit antibodies conjugated with horseradish peroxidase (HRP) were purchased from Santa Cruz Biotechnology.

### RNA Interference

For RNAi gene knockdown experiments targeting the *FLNA* gene, NKL cells were transfected with specific siRNAs (300 pmol) using Amaxa Nucleofector II system (Lonza). Briefly, NKL cells (2 × 10^6^) were resuspended in solution V (100 μL), incubated with the siRNA, and then nucleofected (O-017 program) for a total of 48 h for the assay. For the knockdown of FLNa in primary NK cells, expanded NK cells (1.5 × 10^6^) were resuspended in solution for human macrophages (100 μL), incubated with the siRNAs (300 pmol), and nucleofected (X-001 program). Then, the cells were incubated for 36 h with rIL-2 (200 U/mL), rested for the last 12 h for the assay. To knockdown FLNa in the CD16.NK92 cell line, cells (1.2 × 10^6^) were resuspended in solution R (100 μL), incubated with the siRNAs (300 pmol), and nucleofected (A-024 program) for 48 h. The sequences of the siRNA used to target *FLNA* (siFLNA) are as follows: *FLNA*, 5′- GGC AAA AGU GAC CGC CAA UAA CGA C-3′ (Sense) and 5′- GUC GUU AUU GGC GGU CAC UUU UGC CUC-3′ (Antisense) (38). A second siRNA for *FLNA* was also used with the following sequence: *FLNA*, 5′- GUG ACC GCC AAU AAC GAC AdTdT-3′ (Sense) and 5′- UGU CGU UAU UGG CGG UCA CdTdT-3′ (Antisense) (39). Based on similar results observed with both siRNAs, the results shown are those obtained with the former siRNA oligonucleotides. A negative control siRNA (siControl) was obtained from IDT and Dharmacon.

### Conjugation Assay

Conjugation assay was performed as previously described (18, 40). Briefly, NKL cells are nucleofected with control or *FLNA*-specific siRNAs. Cell tracker green (Invitrogen)-labeled *FLNA*-KD NKL cells were co-incubated with Cell tracker orange (Invitrogen)-labeled 721.221 cells at an effector to target (E:T) ratio of 1:1 for various times. More than 1 × 10^5^ cells were counted using flow cytometry. For analysis, double-positive cells were considered to be conjugated cells following gating on a forward/side scatter.

### Cytotoxicity Assay

The europium-based cytotoxicity assay was used to assess NK cell cytotoxicity. Briefly, target cells were loaded with BATDA reagent (40 μM; Perkin Elmer, Waltham, MA) at 37°C for 30 min. Cells were then washed and incubated with NK cells with sulfinpyrazone at 37°C for the indicated times. The plate was then mixed and centrifuged for 5 min (1400 rpm). Supernatant (20 μL) was then mixed with 20% europium solution (200 μL; Perkin Elmer) in acetic acid (0.3 M) for 5 min, and lysis of target cells was measured with VICTOR X4 multi-label plate reader (Perkin Elmer). For the redirected antibody-dependent cell cytotoxicity (ADCC) assay, FcR+ P815 cells labeled with BATDA reagent were mixed with mAbs (10 μg/mL) raised against the indicated NK receptors at RT for 30 min and then incubated with NK cells in the presence of sulfinpyrazone at 37°C for the indicated times.

### Degranulation Assay

Effector cells were stimulated with the same number of K562, 721.221, or KCL22M cells at 37°C for 2 h. After centrifugation, the cell pellets were resuspended in PBS containing FBS (1%) and stained with anti-CD107a-FITC, anti-CD56-PE, and anti-CD3-PerCP for 30 min at 4°C. For PBMCs, lymphocytes were gated by a forward/side scatter, and CD3-CD56+ cells were defined as NK cells (37, 41). NK cell degranulation, as determined by CD107a on the surface of NK cells, was assessed by flow cytometry.

### Intracellular Cytokine Staining Assay

Effector cells were stimulated with the same number of K562, 721.221, or KCL22M cells at 37°C for 1h. Then, brefeldin A (GolgiPlug; BD Biosciences) was added to block the secretion of cytokines, followed by an extra 5 h of incubation for a total of 6 h. The cells were then surface-stained with anti-CD56-PE and anti-CD3-PerCP for 30 min at 4°C and incubated in BD Cytofix/Cytoperm solution for 20 min at 4°C. After washing with Perm/Wash buffer, the cells were stained intracellularly with anti-IFN-γ-FITC or anti-TNF-α-FITC for 30 min at 4°C and then analyzed using flow cytometry gated on CD3-CD56+ NK cells.

### F-actin Polymerization Assay

To perform an F-actin polymerization assay, NKL cells nucleofected with control siRNA or *FLNA*-specific siRNA were preincubated with isotype control or specific mAbs to NKG2D and 2B4 and then stimulated by receptor crosslinking with goat anti-mouse F(ab′)2 for varying time points. These cells were then fixed, permeabilized, stained with Alexa fluor 488 phalloidin for 2 h, and analyzed by flow cytometry. Changes in the mean fluorescent intensity (MFI) of F-actin were calculated by subtracting the MFI of the unstimulated sample from the MFI of the stimulated sample. Peak fold decreases in F-actin in *FLNA*-KD NKL cells were calculated from the peak F-actin levels observed in Control-NKL and *FLNA*-KD-NKL samples, as previously described (42).

### F-actin Accumulation Assay

In the F-actin accumulation assay, 721.221 target cells were labeled with 2 μM Cell tracker Orange at 37°C for 15 min, and NKL cells and the target cells were co-incubated at an E:T ratio of 1:1 at 37°C for 15 min. Cells were then loaded onto a poly-L-lysine-coated coverslip (Cell Tak 354240, Corning) in a 12 well multi-plate (Nunc). As previously described (43), the cells were then fixed, permeabilized, and stained with Alexa fluor 647 anti-perforin, Alexa fluor 488 phalloidin, or anti-FLNa that was detected by Alexa fluor 647 anti-mouse secondary Ab. Confocal imaging was performed using a 63× objective lens on a Zeiss LSM 710. To assess F-actin localization at the NK IS, images for synaptic F-actin were acquired *via* the z-axis at 0.5 μm fixed intervals from the top and bottom of the cell (e.g., 10~15 images). For quantitative analysis of positive signal, detection settings for control antibody-stained sample was used as a negative control and adjusted to the background levels. To quantify F-actin content at the NK IS, the entire interface between NK and target cell conjugate was covered using a series of 1 μm^2^ square boxes (i.e. three boxes were enough to cover). Thereafter, the mean fluorescent intensity (F-actin MFI) and the area (F-actin area) occupied by phalloidin in the defined region were measured separately in each of the subregions. F-actin content accumulated at the NK IS was obtained by the summation of F-actin area multiplied by the respective F-actin MFI of that particular subregion through the z-axis at 0.5 μm intervals. Furthermore, in an effort to estimate the F-actin content at NK IS as a result of target cell-mediated NK cell activation, the identical three subregions were randomly taken from the plasma membranes of each NK cell and target cell of the same conjugate that are outside of the NK IS. These subregions were measured for F-actin MFI and area as explained above, and their combined value of F-actin content was subtracted from the value of F-actin content accumulated at the NK IS.

### Granule Polarization Assay

The polarization of lytic granules was assessed by microscopy in accordance with previously described protocols (40, 44). Briefly, 721.221 cells were stained with the Cell tracker orange, and co-incubated with NKL cells (1:1 ratio) in serum-free media at 37°C for 30 min to form conjugates. Cells were then transferred to coverslips coated with poly-L-lysine and incubated at 37°C for 30 min. Cells were next fixed in PBS containing formaldehyde (4%), washed twice with PBS, and permeabilized in PBS containing triton X-100 (0.2%), BSA (1%), and sodium citrate (0.1%). After blocking in PBS supplemented with 1% goat serum for 1 h, the cells were washed and incubated with Alexa fluor 488-coupled phalloidin (Invitrogen), and Alexa fluor 647-coupled anti-perforin Ab (Biolegend) for 90 min. Following washing, the coverslips were mounted onto slides using Prolong Gold anti-fade reagent (Molecular Probes). Data were acquired using an LSM 710 laser-scanning microscope (Carl Zeiss). Only conjugates of one NKL cell with one 721.221 cell were analyzed. A minimum of 200 different conjugates was analyzed for each condition. Stages for conjugation were defined as follows: 0, conjugates without granule polarization and actin polymerization; 1, conjugates with actin polymerization, but without polarized granule; 2, conjugates with partial polarization of granules toward the IS; and 3, conjugates with full polarization of granules toward IS.

### Flow Cytometric Ca^2+^ Mobilization Assay

To analyze Ca^2+^ mobilization, NK cells (2 × 10^6^ cells) were loaded with Fluo-4 AM (4 μg/mL) in HBSS (1 mL) containing FBS (1%) and probenecid (4 mM) and incubated for 30 min at 30°C. The cells were then washed twice, resuspended in HBSS supplemented with FBS (1%), and mixed with specific mAbs (10 μg/mL) to the indicated NK receptors for a further 30 min on ice. Cells (5 × 10^5^ cells) were then resuspended in HBSS containing FBS (1%), incubated for 5 min at 37°C, and analyzed by flow cytometry. Following baseline data acquisition (30 s), cells were added with crosslinking goat anti-mouse F(ab′)2 (4 μg), and data were obtained for an additional 5 min. The resulting data were analyzed using FlowJo software (Tree Star).

### Immunoblotting Analysis

Cell lysates of stimulated NK cells were subject to immunoblotting for the indicated proteins, as previously described (31). Protein bands were visualized using SuperSignal West Pico (Pierce) and detected using an LAS-4000 machine (Fujifilm).

### NF-κB-GFP Reporter Assay

NF-κB activation was assessed using NKL-κB-GFP cells, as previously described (31). Briefly, NKL-κB-GFP cells were activated with plate-immobilized specific mAbs to the indicated NK receptors. Then, the reporter cell-mediated GFP expression was assessed using flow cytometry.

### Measurement of NKG2D Internalization

To assess the effect of cognate ligand on surface level of NKG2D receptor by flow cytometry, NKL cells were co-incubated with Far red-labeled P815-ULBP1 cells at a 1:1 ratio in the 96 well V-bottom plate and incubated at 37°C for the indicated times. The cells were then washed with ice-cold DPBS buffer containing 1% FBS and were blocked with Fc Receptor Binding inhibitor (eBioscience) and mouse CD16/CD32 pure antibody (clone; 2.4G2, BD Pharmigen) for 15 min on ice in the dark. Cells were incubated with PE-conjugated anti-NKG2D Ab (clone 149810, R&D system) for 30min on ice in the dark. After washing, the cells were then fixed in PBS containing 2% paraformaldhyde, and washed twice with PBS. Cells were analyzed by flow cytometry using FlowJo software (ver. 10, Tree Star).

To determine the extent of NKG2D internalization by confocal microscopy, NKL cells were first co-incubated with Cell tracker orange CMTMR-labeled P815-ULBP1 cells at a 1:1 ratio on poly-L-lysine-coated coverslips and incubated at 37°C for the indicated times. The cells were then fixed in PBS containing formaldehyde (4%), washed twice with PBS, and permeabilized in PBS with triton X-100 (0.2%), BSA (1%), and sodium citrate (0.1%). After blocking in PBS supplemented with 1% goat serum for 1 h, the cells were washed and incubated with Alexa fluor 647-anti-NKG2D Ab (clone 1D11; Biolegend) for 90 min. After washing, the coverslips were mounted onto slides using Prolong Gold mounting media (Molecular Probes). Data were obtained using an LSM 710 laser-scanning microscope (Carl Zeiss).

### Statistical Analysis

All statistics were calculated using GraphPad Prism software (v. 5.0, GraphPad Software Inc.). Differences between two groups were analyzed using a two-tailed Student’s *t*-test and *P* values < 0.05 were considered significant (*P < 0.05, **P < 0.01, and ***P < 0.001).

## Results

### Colocalization of FLNa With F-Actin at the NK IS

**Figure 1:**
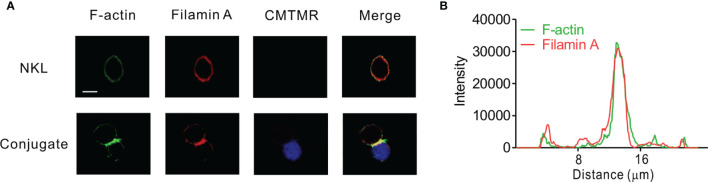
FLNa co-localizes with F-actin at NK IS. **(A)** NKL cells were incubated with Cell tracker orange CMTMR-labeled 721.221 cells (blue) for 30 min. Fixed and permeabilized cells were stained with Alexa 488-coupled phalloidin (green) for F-actin and with anti-FLNa antibody Ab followed by Alexa 647-coupled anti-mouse secondary Ab (red). The images are representative of 10 conjugates analyzed. **(B)** Histograms showing the analysis of green (F-actin) vs red (FLNa) fluorescence intensities as a function of the distance along a line dissecting an NKL-721.221 conjugate through the IS. These curves are representative of five conjugates analyzed. Scale bar, 5 μm. Data are representative of at least three independent experiments.

Given that FLNa is an F-actin cross-linking protein and that F-actin reorganization contributes to lytic IS formation, we first determined the localization of FLNa and F-actin in NKL cells, a human NK cell line, upon their interaction with 721.221 target cells by immunofluorescence microscopy. To facilitate the identification of NKL cells, 721.221 target cells were pre-labelled with Cell tracker orange CMTMR and then incubated with the NKL cells. F-actin polymerization at the lytic IS was imaged by staining with Alexa488-conjugated phalloidin, a highly selective bicyclic peptide that binds F-actin and prevents its de-polymerization (45). FLNa was found to be dispersedly co-localized with F-actin at the perimeter in isolated NKL cells (**Figure 1A**). In comparison, FLNa was concentrated at the IS along with F-actin upon contact of NKL cells with the sensitive 721.221 target cells (**Figure 1A**). To confirm the co-localization of FLNa with F-actin at the IS, we measured the fluorescence intensity of these proteins across the NKL-721.221 conjugate through the IS. We found the highest overlapping spikes in FLNa and F-actin fluorescence intensities at the NKL contact area with 721.221 cells (**Figure 1B**). These results indicate that FLNa is recruited to the IS in NKL cells upon their recognition of sensitive target cells and suggest the potential involvement of FLNa in the formation of the lytic IS.

### FLNa Regulates the Cytolytic Function of NK Cells

**Figure 2:**
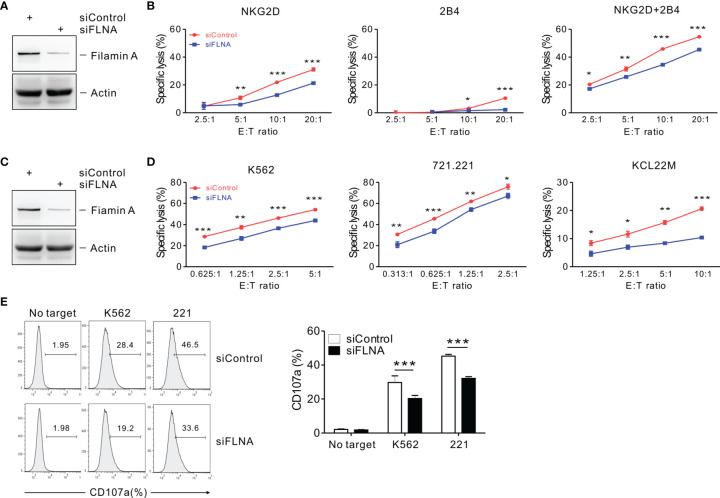
FLNa regulates NK cell cytotoxicity. **(A)** Lysates of NKL cells nucleofected with a control siRNA or FLNa-specific siRNA were immunoblotted for FLNa and actin. **(B)** P815 cell lysis by NKL cells nucleofected with a control siRNA or siRNA specific for FLNa at the indicated E:T ratio. The cytotoxicity was then measured against P815 cells preincubated with specific mAbs to NKG2D, 2B4, or their combination after 2 h using the Europium assay. **(C)** Total lysates of CD16.NK92 cells nucleofected with control siRNA or siRNAs specific for FLNa were immunoblotted for FLNa and actin. **(D)** Lysis of K562, 721.221, or KCL22M cells by CD16.NK92 cells nucleofected with control siRNA or FLNa-specific siRNA at the indicated E:T ratio. The cytotoxicity against K562, 721.221, or KCL22M cells was measured using 2 h-europium release assay. **(E)** CD16.NK92 cells nucleofected with control siRNA or siRNA specific for FLNa were mixed with K562 or 721.221 cells with labeled anti-CD107a to conduct a degranulation assay for 2 h. Thereafter, the cells were surface stained with labeled anti-CD56 mAbs. CD107a expression on CD16.NK92 cells was determined on CD56+ cells by flow cytometry. Representative FACS profiles (*left*) and a summary graph (*right*) of the expression of CD107a on CD16.NK92 cells are shown. Error bars represent the SD; *P < 0.05, **P < 0.01, ***P < 0.001. Data are representative of at least three independent experiments.

**Figure 3:**
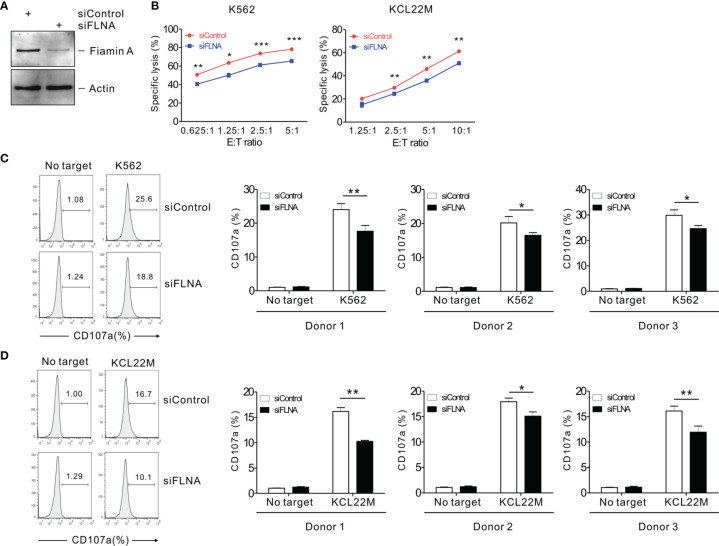
FLNa regulates the cytotoxicity of primary NK cells. **(A)** Lysates of primary expanded NK cells nucleofected with control or FLNa-specific siRNAs were assessed by immunoblotting for FLNa and actin. **(B)** Lysis of K562 cells or KCL22M cells by primary expanded NK cells nucleofected with control or FLNa-specific siRNAs at the indicated E:T ratio. Cytotoxicity against K562 or KCL22M cells was determined using 1 h-europium release assay. **(C, D)** Primary expanded NK cells nucleofected with control or FLNa-specific siRNAs were incubated with K562 **(C)** or KCL22M cells **(D)** with labeled anti-CD107a for 2 h degranulation assay. Then, the cells were surface-stained with labeled anti-CD56 mAb. CD107a expression by primary expanded NK cells was determined on CD56+ cells by flow cytometry. Representative FACS profiles (*left*) and summary graphs (*right*) indicating the expression of CD107a on primary expanded NK cells from three different healthy donors are shown. Error bars represent the SD; *P < 0.05, **P < 0.01, ***P < 0.001. Data are representative of three independent experiments.

To investigate the effects of FLNa on NK cell cytotoxicity, we silenced the expression of FLNa using siRNA-mediated knockdown (KD) (**Figure 2A**). NKL cells depleted of FLNa were then triggered by different activating receptors such as NKG2D and/or 2B4 through the stimulation with FcR+ P815 target cells coated with antibodies specific to these receptors (31). NKG2D and 2B4 are key NK cell activating receptors, with well-established ligands and signaling pathways (6). The combined stimulation of NKG2D and 2B4 has been shown to trigger cytotoxic degranulation in a synergistic manner in resting primary NK cells and only NKL cell line among the cell lines NK92, NKL, NK3.3, and YTS tested (41, 46). The FLNa KD caused significant decrease in the cytotoxicity of NKL cells in response to NKG2D and 2B4, both alone and in combination (**Figure 2B**), suggesting an involvement of FLNa in the regulation of NK cell cytotoxicity by different activating receptors. To ascertain whether FLNa is also required for cytotoxicity by different NK cell line, NK92 cells, a highly cytotoxic human NK cell line, were next co-incubated with three different types of target cells: K562, 721.221, and KCL22M cells. Following the FLNa KD in NK92 cells (**Figure 2C**), their cytotoxicity was significantly impaired in response to all three target cell types (**Figure 2D**). Consistently, cytotoxic degranulation, as measured by CD107a expression on NK92 cells, was significantly decreased by FLNa KD in response to K562 and 721.221 cells (**Figure 2E**). Hence, using different NK cell lines, we have found evidence that FLNa is required for the NK cell cytotoxic response in response to various target cells that engage different activating receptors. We next investigated the effects of FLNa on the regulation of cytotoxicity in primary NK cells. At 48 h after transfection with the FLNa KD siRNA, we observed a noticeable silencing of FLNa in primary expanded NK cells (**Figure 3A**). The FLNa KD resulted in a significant impairment of natural cytotoxicity of NK cells against K562 and KCL22M cells (**Figure 3B**), correlating with cytotoxic degranulation against these target cells (**Figures 3C, D**). Taken together, these results indicate that FLNa is required for NK cell-mediated cytotoxicity.

### FLNa Is Required for NK-Target Interaction

**Figure 4:**
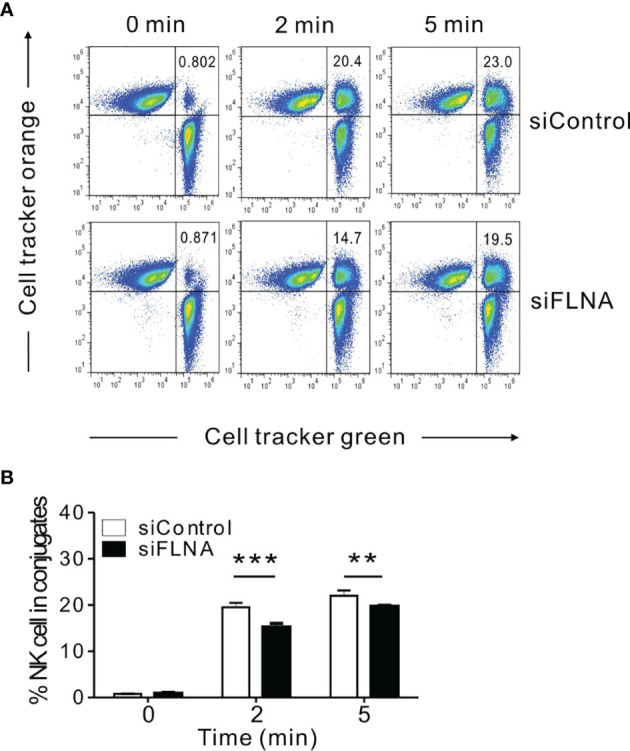
FLNa is required for conjugate formation between NK and target cells. **(A, B)** NKL cells nucleofected with control or FLNa-specific siRNAs were loaded with Cell tracker green and incubated with Cell tracker orange-labeled 721.221 target cells at an E:T ratio of 1:1 for the indicated times. Thereafter, the cells were fixed and then analyzed by flow cytometry for detecting conjugate formation, as assessed by the double-positive population (upper right quadrant). Shown are representative results **(A)** and a summary graph **(B)**. Error bars represent the SD; **P < 0.01, ***P < 0.001. Results are representative of three independent experiments.

To better understand the mechanisms underlying the regulation of NK cell cytotoxicity by FLNa, we investigated the effects of FLNa on a series of steps required for this cytotoxic response. An initial step in the formation of the lytic IS includes the close association between NK cells and their target cells, followed by their firm adhesion *via* the integrin family of adhesion molecules (8). Such a tight interaction is important for actin reorganization, downstream signaling by NK activating and inhibitory receptors, and for the targeted polarization and secretion of lytic granules. Hence, we first conducted a cell conjugation assay to investigate whether FLNa affects conjugate formation between NK and target cells. To facilitate their identification, NKL cells were labeled with Cell tracker green CMFDA and then incubated with Cell tracker orange-labeled 721.221 cells to allow conjugate formation for different time periods. FLNa KD significantly impaired the ability of NKL cells to form conjugates with 721.221 cells, by approximately 28% and 15% at 2 min and 5 min, respectively (**Figures 4A, B**), suggesting that FLNa is required for NK cell-target cell conjugate formation.

### FLNa Enhances the F-actin Density at the NK IS Without Affecting Granule Polarization

**Figure 5:**
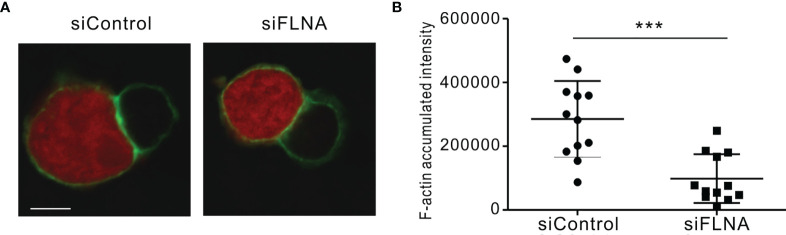
F-actin density reduction by an FLNa deficiency at the NK IS. **(A)** NKL cells nucleofected with control or FLNa-specific siRNAs were incubated with Cell tracker orange CMTMR-loaded 721.221 cells (red) for 15 min, fixed, permeabilized, and stained for F-actin (green). F-actin accumulation at the IS between NKL and 721.221 cells was analyzed by confocal imaging. The image is representative of 12 conjugates analyzed. **(B)** F-actin accumulation at the NK IS was estimated using a reconstruction of serial confocal images acquired *via* the z-axis and a single compressed image of the NKL and 721.221 cells conjugate shown in **(A)**. A summary graph is presented. The horizontal line represents the mean, and the vertical bar indicates the SD; ***P < 0.001. Scale bar, 5 μm. Results are representative of three independent experiments.

We next investigated the effects of FLNa depletion on the accumulation of F-actin at the NK IS and on lytic granule polarization to determine the extent to which it impacts on the sequential steps required for NK cytotoxicity. In the experiment, NKL cells depleted of FLNa were allowed to form conjugates with Cell tracker orange-labeled 721.221 cells, were stained for F-actin with phalloidin, and then imaged for F-actin accumulation at the synapse by confocal microscopy. FLNa-deficient NK cells had a decreased F-actin density at the IS upon contact with target cells, as determined by the estimation of the total F-actin content at the NK IS based on the accumulated intensity of phalloidin fluorescence, although we could not completely rule out the contribution of F-actin content from target cells (**Figures 5A, B**). To next determine whether the defect in F-actin reorganization was an intrinsic feature of FLNa-deficient NK cells, we assessed the effect of FLNa depletion on activation-induced F-actin polymerization in NK cells without target cells. To this end, NKL cells were stimulated by preincubation with anti-NKG2D and anti-2B4 antibodies followed by cross-linking with anti-mouse F(ab′)2 fragment. The results revealed a significant reduction in the F-actin content in FLNa-deficient NKL cells upon activation but not in their basal state (**Supplementary Figure S1**). This was compatible with a defect in F-actin accumulation at the NK IS upon stimulation with target cells. These findings thus suggest that FLNa is required for the F-actin polymerization induced by activation-induced signaling *via* NKG2D and 2B4 receptors, as well as through target cell contact.

F-actin accumulation at the NK IS is followed by the targeted polarization of converged lytic granules along with the MTOC toward the synapse (47, 48). We therefore assessed the level of cytotoxic granule polarization in NK cells conjugated with Cell tracker orange-loaded 721.221 cells by confocal microscopy. Cytolytic granules along with F-actin accumulation at the NK IS were detected by anti-perforin and phalloidin staining, respectively. Granule polarization was scored by assessing the different conjugate stages in accordance with the progression of polarized granules toward the lytic synapse. Upon conjugate formation with target cells (stage 0), F-actin is accumulated at the IS in stage 1, which is followed by the partial and then full polarization of perforin (red)-containing granules toward the NK IS in stages 2 and 3, respectively (**Supplementary Figures S2A, B**). Of note, the measurement of granule polarization was found to be unaffected by the loss of FLNa at all of the conjugate stages tested (**Supplementary Figures S2C, D**). These results indicated that a defect in lytic IS formation, but not in granule polarization, could be a potential mechanism underlying the observed cytotoxic dysfunction of FLNa-deficient NK cells.

### FLNa Restrains NK Cell Cytokine Production

**Figure 6:**
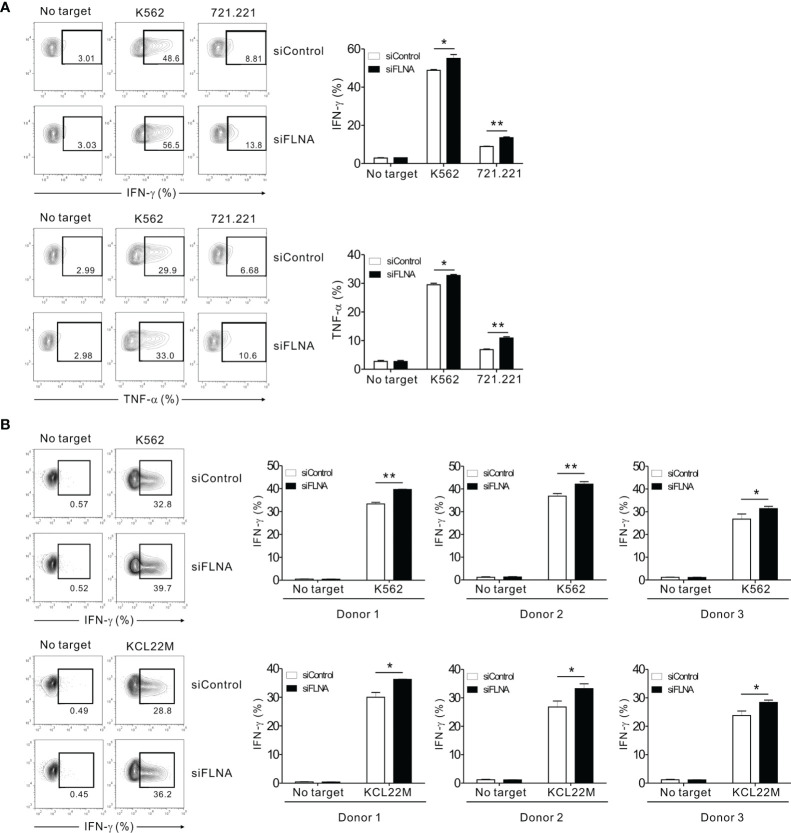
FLNa suppresses cytokine production by NK cells. **(A)** CD16.NK92 nucleofected with control siRNA or FLNa-specific siRNA were incubated with K562 or 721.221 cells for a total of 6 h to conduct an intracellular cytokine staining assay. Then, the cells were surface stained with labeled anti-CD56 mAbs. NK cell cytokine production was assessed among the CD56+ cells by flow cytometry with intracellular staining for IFN-γ and TNF-α. Representative FACS profiles (*left*) and a summary graph (*right*) showing the expression of IFN-γ and TNF-α in CD16.NK92 cells are presented. **(B)** Primary expanded NK cells nucleofected with control or FLNa-specific siRNAs were incubated with K562 or KCL22M cells for 6 h. Thereafter, the cells were surface stained with a labeled anti-CD56 mAb. NK cell cytokine production was assessed among the CD56+ cells by flow cytometry with intracellular staining of IFN-γ. Representative FACS profiles (*left*) and summary graphs (*right*) showing the expression of IFN-γ in primary expanded NK cells from three different healthy donors are presented. Error bars represent the SD; *P < 0.05, **P < 0.01. Results are representative of three independent experiments.

We next investigated the effects of FLNa depletion on cytokine production by NK cells as this is a pivotal component of the innate immune response, particularly the expression of IFN-γ and TNF-α (49). The intracellular expression of IFN-γ and TNF-α was assessed in NK92 cells in response to K562 and 721.221 cells. Unexpectedly, the FLNa KD led to a significant enhancement of IFN-γ and TNF-α expression in NK92 cells following stimulation with the target cells (**Figure 6A**). Using primary expanded NK cells, we also observed a significant increase in IFN-γ expression following an FLNa deficiency in response to K562 and KCL22M cells (**Figure 6B**). Consistently, IFN-γ and MIP-1α production from NKL cells, as measured by ELISA, were significantly increased by FLNa KD in response to 721.221 cells (**Supplementary Figure S3**) despite a significant decrease in cytotoxicity against 721.221 cells by FLNa KD (data not shown). Our results thus suggest that FLNa restrains NK cell cytokine production in response to sensitive target cells, and that this is distinct from its requirement for NK cell cytotoxicity.

### FLNa Regulates Calcium-Dependent Signaling and NF-κB Activation in NK Cells

**Figure 7:**
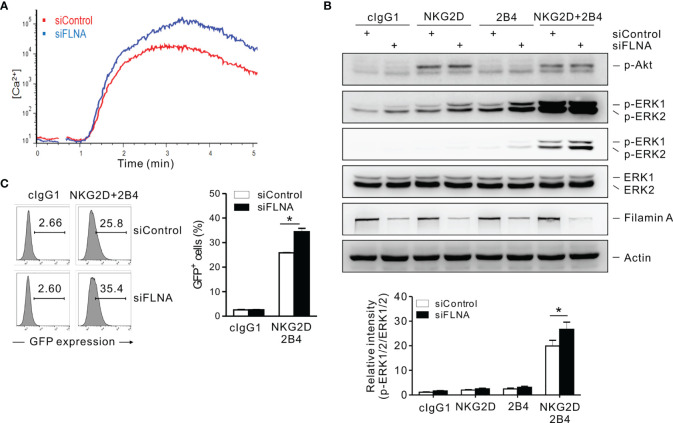
An FLNa knockdown potentiates both calcium-mediated signaling and NF-κB activity. **(A)** Rested NKL cells nucleofected with control or FLNa-specific siRNAs were labeled with Fluo-4 for 30 min at 30°C and stimulated *via* NKG2D and 2B4 after measuring the baseline Ca^2+^ flux for 30 s. Ca^2+^ mobilization-mediated changes in fluorescence are shown as a function of time. **(B)** Rested NKL cells nucleofected with control or FLNA-specific siRNAs were preincubated with an isotype control or with specific mAbs to NKG2D and/or 2B4 and stimulated by receptor crosslinking for 2 min. Lysates were subject to immunoblotting for p-Akt, p-ERK1/2, ERK1/2, FLNa, and actin (*top*). Band intensities of phosphorylated ERK1/2 relative to total ERK1/2 were quantified using ImageJ software and are presented (*bottom*). **(C)** Rested NKL cells transduced with a κB reporter construct were stimulated with plate-immobilized mAbs against NKG2D and 2B4 for 6 h. GFP expression in NKL-κB-GFP cells was analyzed by flow cytometry, and representative results (*left*) and a summary graph (*right*) are shown. Error bars represent the SD; *P < 0.05. Data are representative of three independent experiments.

A calcium-mediated signaling pathway that leads to ERK activation is indispensable for the effector functions of NK cells in response to target cells (37, 41, 50). The stimulation of NKL cells *via* a combination of NKG2D and 2B4 was found to trigger a robust Ca^2+^ influx, which was augmented by the loss of FLNa (**Figure 7A**). The phosphorylation of ERK, downstream of calcium-dependent signaling, rather than Akt was also enhanced by an FLNa deficiency, which was only significant upon NKG2D and 2B4 stimulation (**Figure 7B**). Moreover, NF-κB activation, as assessed with reporter cells (NKL-κB-GFP), was also significantly increased in FLNa-deficient NK cells upon NKG2D and 2B4 stimulation (**Figure 7C**), consistent with the contribution of ERK to NF-κB activation in NK cells (31). These results suggest therefore that FLNa negatively regulates calcium-dependent signaling and suppresses ERK and NF-κB activation in NK cells, which correlates with its regulation of cytokine production, such as IFN-γ.

### FLNa Promotes NKG2D Receptor Internalization

**Figure 8:**
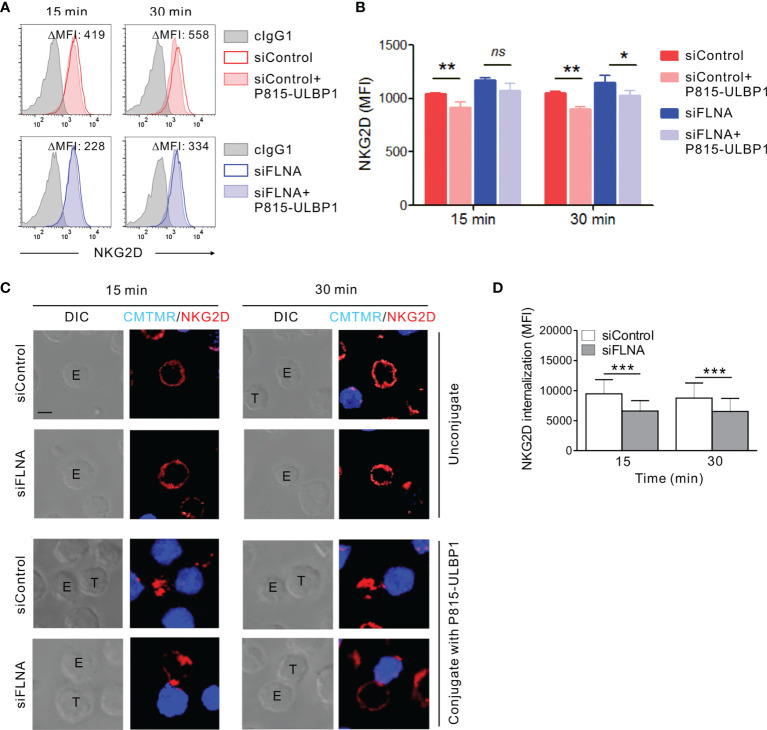
Loss of FLNa attenuates ligand-induced NKG2D internalization. **(A, B)** Rested NKL cells nucleofected with control siRNA or FLNa-specific RNA were incubated with Far red-labeled P815-ULBP1 cells for the indicated times. The surface levels of NKG2D on NKL cells were measured by flow cytometry after staining with an anti-NKG2D Ab. Representative FACS plots **(A)** and statistical graph **(B)** showing the mean fluorescent intensity (MFI) of surface NKG2D after co-culture with P815-ULBP1 cells relative to NKG2D MFI without co-culture (ΔMFI). **(C)** Rested NKL cells nucleofected with control or FLNa-specific siRNAs were incubated with Cell tracker orange CMTMR-labeled P815-ULBP1 cells (blue) for the indicated times. Conjugates were subject to fix and permeabilization and then stained with an anti-NKG2D Ab (red). Conjugates were analyzed using confocal microscopy to assess the localization of NKG2D in the NKL cells. Representative confocal images of NKL cells (*top*) or conjugates between NKL cells and P815-ULBP1 cells (*bottom*) are presented. **(D)** Quantification of NKG2D internalization in NKL cells conjugated with P815-ULBP1 cells. Error bars represent the SD; *P < 0.05, **P < 0.01, ***P < 0.001. Scale bar, 5 μm. Results are representative of three independent experiments. ns, not significant.

We next investigated whether the augmentation of activation signals involving NKG2D caused by an FLNa deficiency could be attributed to a defect in the regulation of the NKG2D receptor. It is known that the engagement of NKG2D with its cognate ligands, such as MICB, triggers NK cell activation and in turn the downregulation of receptor surface expression for lysosomal degradation (51, 52). Given the role of FLNa in the regulation of various receptor internalization including the chemokine receptor CCR2B (53–56), ligand-induced NKG2D downregulation was evaluated by flow cytometry in FLNa-deficient NK cells in the presence of target cells expressing ULBP1, a NKG2D ligand. NKL cells depleted of FLNa were incubated alone or in the presence of P815 cells expressing ULBP1 for various times and then analyzed by FACS to assess the levels of surface NKG2D. As expected, a significant reduction in surface NKG2D was observed in NKL cells cultured with P815-ULBP1 cells (**Figures 8A, B**). Of note, such a decrease in the level of surface NKG2D was insignificant at 15 min and less significant after 30 min of incubation by FLNa deficiency besides an increased basal level of surface NKG2D (**Figures 8A, B**). To confirm this finding in the context of single cell level, ULBP1-induced NKG2D internalization was then studied by confocal microscopy. NKL cells depleted of FLNa were incubated with Cell tracker orange-loaded P815-ULBP1 cells for various times. NKG2D was observed to be evenly distributed at the perimeter of isolated NKL cells (**Figure 8C**). By contrast, NKG2D was downregulated at the surface of NK cells conjugated with P815-ULBP1 target cells, but this response was significantly impaired in FLNa-deficient NK cells, as determined by the mean fluorescence intensity of the NKG2D redistributed as intracellular dots in randomly acquired fields of 40 NKL and P815-ULBP1 conjugates (**Figures 8C, D**). These results suggest that interaction with ULBP1-expressing target cells induces NKG2D internalization in NK cells, and that this is impeded by the loss of FLNa.

## Discussion

The appropriate regulation of the actin cytoskeleton between NK cells and their target cells is indispensable for the efficient NK cell-mediated cytolysis and is largely dependent on actin regulatory factors involving Arp2/3 complex and the formins, WASps family of proteins, and Coronin 1A (7). Despite their critical and well-defined contribution to this cytotoxic response through the mediation of actin polymerization, de-polymerization, and branching, the role of actin cross-linking/bundling for F-actin network formation mediated by filamins remains unexplored in this context. We here demonstrated that FLNa, a major isoform of filamin expressed in NK cells (57), is required for NK-target cell conjugate formation and F-actin accumulation at the NK IS, leading to the efficient cytolysis of the target cells. The effect of FLNa on NK cell cytotoxicity did not appear to depend on specific NK activating receptors or the target cells tested herein that engage different signaling pathways. We also observed consistent results with different NK cell lines and primary NK cells from multiple donors, supporting the requirement of FLNa for NK cell cytotoxicity. Surprisingly, an FLNa deficiency did not impair but augmented the NK cell expression of IFN-γ and TNF-α, correlating with an increase in activation signals such as Ca^2+^ mobilization, ERK, and NF-κB and a defect in the downregulation of the NKG2D receptor. These findings support the notion that FLNa contributes to the dichotomous regulation of NK cell cytotoxicity and cytokine production for the triggering of optimal effector functions.

There have been multiple reports suggesting that the pathway leading to NK cell cytotoxicity *via* cytotoxic granules is uncoupled to, and distinct from, that for cytokine secretion (58, 59). While perforin-containing granules are delivered and released in a polarized fashion at the contact site between NK cells and target cells, cytokines such as IFN-γ and TNF-α are delivered all over the cell surface, including the NK IS, *via* recycling endosomes for principally nonpolarized release (58). In addition, NK cells from patients with Chediak-Higashi syndrome entailing mutations in the lysosomal trafficking regulator exhibit severely impaired cytotoxicity but have a normal capacity for cytokine secretion (59). This separation of regulatory pathways is considered an important mechanism enabling NK cells to recruit and activate other immune cells *via* cytokine secretion, in addition to the effective killing of target cells. From the perspective of signaling processes, a PI3K p110δ deficiency has been shown previously to impair cytokine secretion but not cytotoxicity (60), whereas Vav1 is known to be required for cytotoxicity but not for IFN-γ production in mouse NK cells (61). Based on our current findings of the reciprocal regulation of NK cell cytotoxicity and cytokine production by FLNa, we further speculate that NK cells become committed to killing target cells once they encounter them, while at the same time limiting the production of cytokines *via* FLNa-mediated F-actin reorganization. This interdependent regulation between cytotoxicity and cytokine production by actin cytoskeleton was hinted by previous studies of the effect of actin polymerization inhibitors on the effector functions of T cells and NK cells (62, 63). Disruption of actin polymerization by cytochalasin D (CCD) or latrunculin B (Lat B) results in impaired T cell-APC conjugate formation but potentiates intracellular Ca^2+^ mobilization, NFAT nuclear duration, and cytokine production including IL-2 in response to anti-CD3, thapsigargin, or phorbol myristate acetate plus ionomycin (62). Of note, pharmacological inhibition of actin polymerization by low doses of CCD, Lat B, or Lat A leads to the activation of human NK cells rather than T cells in terms of IFN-γ and TNF-α production in response to anti-CD16 or IL-12 (63), despite an inhibitory effect of CCD on NK cell cytotoxicity (64). In support, we also observed the reciprocal regulation of NK cell cytotoxicity and IFN-γ production upon treatment with low doses of CCD and Lat B (data not shown). Moreover, a recent study has reported FLNa as a new regulator of NK cell migration, showing that an FLNa deficiency increases the migration of human NK cell lines (57). Consistently, another study has found that increased FLNa binding to β-integrin tails restricts cell migration (65). Hence, it is likely that FLNa may help to coordinate tailored NK cell responses during the NK cell decision to kill target cells by regulating migration as well as effector functions. The mechanisms underlying this coordination and the regulation of FLNa levels in a pathophysiological context are not yet clear and merit further investigation.

It has been established that NK cell adhesion to target cells is followed by the formation of a lytic synapse and the polarized delivery of cytotoxic granules toward the NK IS (17, 47, 48, 66). We revealed in our present analyses that FLNa regulates NK-target cell conjugate formation, F-actin accumulation at the IS, degranulation, and cytotoxicity, but not cytolytic granule polarization. These results suggest that the signaling pathways regulated by FLNa are uncoupled from those that function in granule polarization. Three key components of the cytoskeleton have been proposed in terms of the division of labor for NK cell cytotoxicity: actin for adhesion, IS formation, and signal transduction, non-muscle Myosin II for the degranulation of lytic granules, and microtubule for the polarized delivery of lytic granules along with the MTOC (7). Hence, it was not unexpected in our present experiments that granule polarization toward a bound target cell was intact in the absence of FLNa-mediated F-actin accumulation at NK IS. In line with our results, the polarization of MTOC is responsible for the polarization of cytolytic granules, but to be dispensable for IS formation in NK cells (67).

Filamins are elongated homodimeric proteins that crosslink F-actin filaments and function as membrane scaffolds for receptor anchoring, trafficking, and certain signaling pathways (68, 69). We demonstrated in our present study that F-actin cross-linking/bundling mediated by FLNa is required for NK cell-mediated cytotoxicity. Besides filamins, fimbrin/plastin and α-actinin are also F-actin cross-linking proteins (26). A recent study reported that L-plastin is involved in NKG2D clustering into lipid rafts but does not significantly affect NKG2D-mediated functions such as degranulation and IFN-γ expression in NK cells (70). In our current analyses however, FLNa was found to be involved in NKG2D internalization, supporting the notion that the internalization of NKG2D and subsequent signal transduction require the proper regulation of F-actin cross-linking/bundling. We speculate that the sustained presentation of NKG2D in relation to its impaired internalization caused by an FLNa deficiency may underlie the upregulation of activation signals such as Ca^2+^ mobilization, ERK, and NF-κB in NK cells observed in our current study. In support of this possibility, a recent study has demonstrated that FLNa is required for the initial step in chemokine CCR2B receptor endocytosis after ligand stimulation. Accordingly, an FLNa deficiency leads to the delayed internalization of the chemokine CCR2B receptor (53). Despite this enhanced activation and cytokine production, we observed an impaired degranulation and a reduced cytotoxicity of FLNa-deficient NK cells against target cells. As an efficient cytotoxicity by NK cells requires combined signals for adhesion, granule polarization, and degranulation (71), we postulate that a defective conjugate formation and disrupted F-actin accumulation at the NK IS could be a potential mechanism underlying the cytotoxic dysfunction of FLNa-deficient NK cells. Supporting this notion, the release of granzyme B, a key cytotoxic effector molecule under the control of NF-κB activation (31), was modestly increased in FLNa-deficient NKL cells upon stimulation with beads coated with anti-NKG2D and anti-2B4 antibodies without target cells (**Supplementary Figure S4**). Given an impaired cytotoxicity of FLNa-deficient NK cells, this discrepancy between granzyme B release and lytic effector function was compatible with a requirement of proper conjugate formation between NK and target cells for activating receptors to trigger cytotoxicity (46, 71). Moreover, although significant, the relatively modest effect of FLNa deficiency suggests that additional actin regulators could be involved in such context and might partially compensate for the loss of FLNa. Consistently in this regard, a loss of Arp2/3 downstream of LFA-1 and NKG2D has been shown to lead to defects in adhesion and actin assembly at the lytic synapse, culminating in the reduced cytolytic activity of NK cells (20, 72). Hence, as a crucial determinant of the stiffness of F-actin networks for cell adhesion and migration (59, 73), it is possible that FLNa cooperates simultaneously or sequentially with other actin-binding proteins such as Arp2/3 (25, 68) to bundle the F-actin cytoskeleton and thereby maintain actin dynamics at the NK IS, ultimately resulting in optimal NK cell cytotoxicity. Future studies are needed to address the molecular mechanisms underlying this cooperative process with respect to the regulation of cytotoxicity and cytokine production.

In summary, FLNa has a previously unappreciated role in the regulation of NK cell effector functions beyond its defined inhibitory effects on the migration of NK cells (57). FLNa was found to be required for conjugate and lytic IS formation, degranulation, and the eventual NK cell-mediated cytolysis of target cells, whilst limiting the production of cytokines such as IFN-γ and TNF-α. The primary function of FLNa is to cross-link actin filaments with orthogonal orientation in response to various stimuli, which regulates the mechanical stiffness of the F-actin network in a concentration-dependent manner (73–75). In this regard, it is tempting to speculate that F-actin cross-linking/bundling mediated by FLNa is a necessary process coordinating optimal NK effector functions in addition to the contribution of F-actin polymerization, branching, and deconstruction.

## Data Availability Statement

The original contributions presented in the study are included in the article/**Supplementary Material**. Further inquiries can be directed to the corresponding authors upon reasonable request.

## Ethics Statement

Human primary samples were obtained from healthy donors after informed consent in accordance with protocols approved by the Institutional Review Board (IRB) of Asan Medical Center and the Declaration of Helsinki. The patients/participants provided their written informed consent to participate in this study.

## Author Contributions

NK, EY, SK, HP, and H-JK were involved in data acquisition. NK, EY, and HK were involved in literature review, data analysis, and interpretation. NK, EY, SK, and HK contributed to the conceptual design of the study and writing of the manuscript with an input from all co-authors. All authors contributed to the article and approved the submitted version.

## Funding

This study was supported by the National Research Foundation of Korea (NRF) grant funded by the Korea government (MSIT) (2019R1A2C2006475, 2020R1I1A1A01069754), MRC grant (2018R1A5A2020732) funded by the Korea government (MSIT), grant from the Korea Healthy Technology R&D Project, Ministry of Health & Welfare (HI21C1568), and partly by a grant (2021IP0003, 2021IL0013) from the Asan Institute for Life Sciences, Asan Medical Center, Seoul, Korea.

## Conflict of Interest

The authors declare that the research was conducted in the absence of any commercial or financial relationships that could be construed as a potential conflict of interest.

## Publisher’s Note

All claims expressed in this article are solely those of the authors and do not necessarily represent those of their affiliated organizations, or those of the publisher, the editors and the reviewers. Any product that may be evaluated in this article, or claim that may be made by its manufacturer, is not guaranteed or endorsed by the publisher.
