# Supplementary material for: Filamin A Is Required for NK Cell Cytotoxicity at the Expense of Cytokine Production via Synaptic Filamentous Actin Modulation
Source: Front Immunol. 2022 Jan 4;12:792334. doi: 10.3389/fimmu.2021.792334 (PMC8764188; doi:10.3389/fimmu.2021.792334)
Supplement: Supplementary file 1 [file DataSheet_1.docx]

Supplementary Material

**Filamin A is required for NK cell cytotoxicity at the expense of cytokine production via synaptic filamentous actin modulation**

Nayoung Kim^1#^, Eunbi Yi^2#^, Soon Jae Kwon^2^, Hyo Jin Park^2^, Hyung-Joon Kwon^2^ and Hun Sik Kim^3,*^

^1^Department of Convergence Medicine, Asan Institute for Life Sciences, Asan Medical Center, University of Ulsan College of Medicine, Seoul 05505, Republic of Korea

^2^Department of Biomedical Sciences, Asan Medical Center, University of Ulsan College of Medicine, Seoul 05505, Republic of Korea

^3^Stem Cell Immunomodulation Research Center (SCIRC), University of Ulsan College of Medicine, Seoul 05505, Republic of Korea

#These authors have contributed equally to this work

*Correspondence: Prof. Hun Sik Kim, Department of Biomedical Sciences, Asan Medical Center, University of Ulsan College of Medicine, 88 Olympic-ro 43-gil, Songpa-gu, Seoul 05505, Republic of Korea. E-mail: hunkim@amc.seoul.kr

**Supplementary figures**

**
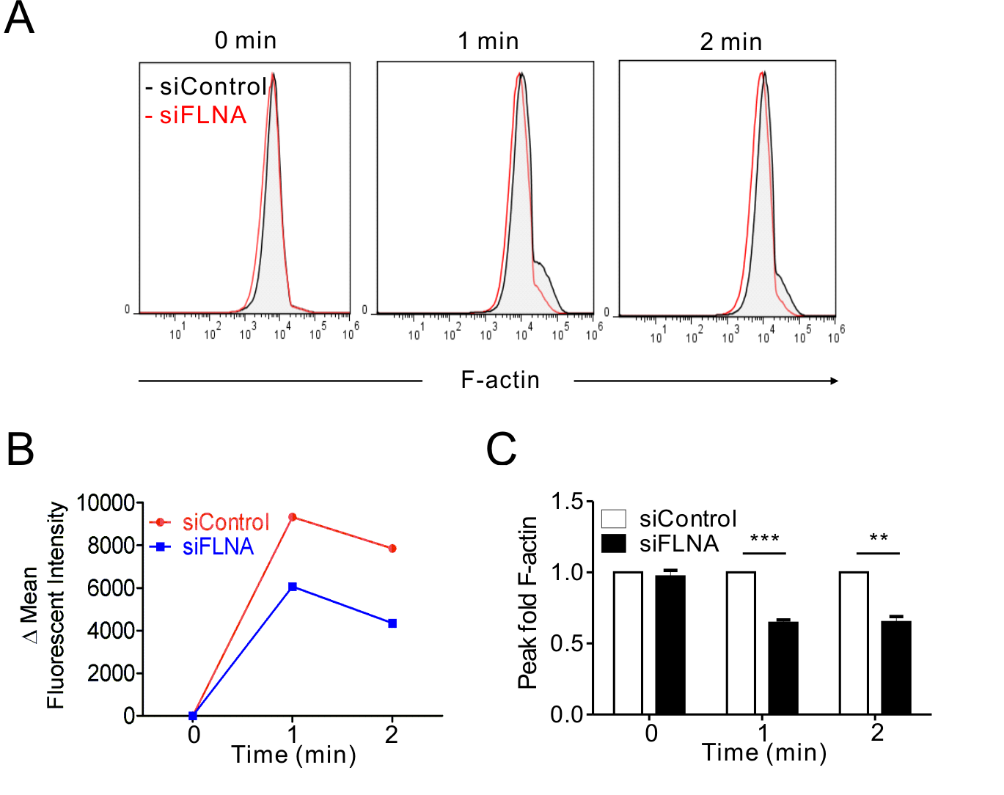
**

**Figure S1.** ***FLNA* KD decreases actin polymerization in NKL cells.**

(**A**, **B**) NKL cells transfected with control siRNA or FLNa-specific siRNA were preincubated with isotype control mAb or mAbs specific for NKG2D and 2B4 and were then stimulated by receptor crosslinking for the indicated time point. NKL cells were fixed, permeabilized, stained with Alexa 488-coupled phalloidin for F-actin, and then analyzed by flow cytometry. Changes in mean fluorescent intensity (MFI) of F-actin were calculated by subtracting MFI of unstimulated samples from the MFI of stimulated samples (ΔMFI). Shown are representative FACS profiles (A) and line charts (B). (**C**) Summary graph showing the fold changes of F-actin calculated from peak levels in each group. Error bars represent the SD. ***P* < 0.01; ****P* < 0.001. Data are representative of three independent experiments.


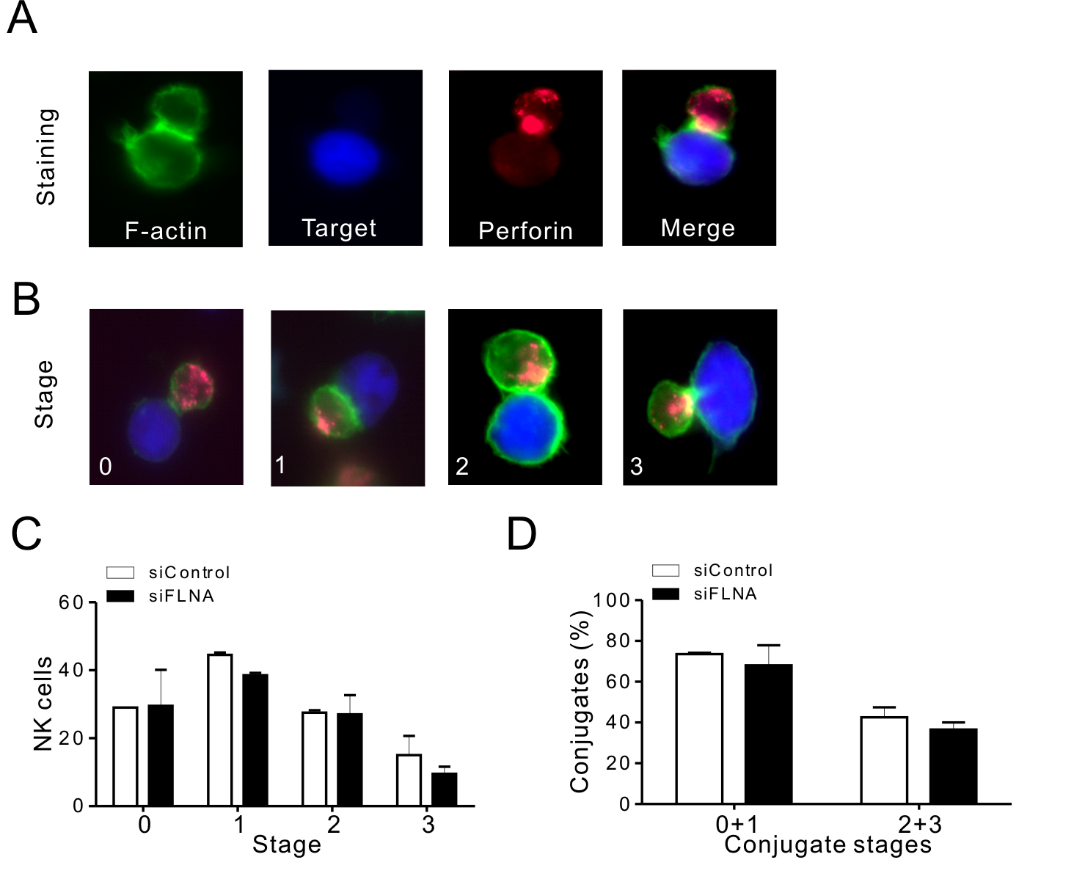


**Figure S2.** **FLNa does not affect granule polarization.**

(**A**, **B**) Representative confocal images of conjugates between NKL cells and Cell tracker orange CMTMR-labeled 721.221 cells (blue). Conjugates were fixed, permeabilized, and then stained with phalloidin (F-actin; green) and anti-perforin Ab (red). Conjugates were analyzed by confocal microscopy to determine the polarization of perforin-containing granules toward target cells (A). Conjugates were categorized into different stages according to the progression of granule polarization toward target cells. Shown are conjugates representative of each stage (B). (**C**) NKL cells transfected with control siRNA or FLNa-specific siRNA were incubated with Cell tracker orange CMTMR-loaded 721.221 cells for 15 min. Cells were stained as described in (A), and percentages of NKL cells at each stage of granule polarization were measured with at least 100 NKL-target cell conjugates. Shown are summary graph from three independent experiments. (**D**) Data from (C) were analyzed after changing four conjugate stages into two-related conjugate stages.

**

**

**Figure S3. Loss of FLNa enhances cytokine production of NK cells.**

(**A**, **B**) NKL cells transfected with control siRNA or FLNa-specific siRNA were stimulated with 721.221 cells for 6 h. Then, IFN-γ (A) and MIP-1α (B) in the supernatants were measured by ELISA. Error bars represent the SD. ****P* < 0.001. Data are representative of three independent experiments.

**

**

**Figure S4. Loss of FLNa increases NK cell release of granzyme B.**

(**A**, **B**) NKL cells transfected with control siRNA or FLNa-specific siRNA were stimulated with beads coated with isotype control mAb or mAbs specific for NKG2D and 2B4. After 2 h incubation, granzyme B in the supernatants were measured by ELISA. Error bars represent the SD. **P* < 0.05. Data are representative of two independent experiments.
